# Community-associated Methicillin-resistant *Staphylococcus aureus*

**DOI:** 10.3201/eid1209.060162

**Published:** 2006-09

**Authors:** Bala Hota, Robert A. Weinstein

**Affiliations:** * Rush University Medical Center, Chicago, Illinois, USA;; † John H. Stroger Jr. (Cook County) Hospital, Chicago, Illinois, USA

**Keywords:** Surveillance, Informatics, *Staphylococcus aureus*, letter

**To the Editor:** Community-associated (CA) methicillin-resistant *Staphylococcus aureus* (CA-MRSA) is a global emerging threat (*1**–**7*). Accurate measures of the extent of CA-MRSA are critical to allocate resources, guide control measures, and inform prescribing practices (*8*). We assessed the utility of administrative databases, a computerized clinical data repository, and an electronic rule to enhance surveillance for CA-MRSA at Stroger (Cook County) Hospital, a 464-bed public safety net hospital in Chicago, and its associated clinics—all part of the Cook County Bureau of Health Services (CCBHS).

Using data collected within the Chicago Antimicrobial Resistance Project computerized clinical data repository (*9*) from September 1, 2001, to August 31, 2004, we developed an electronic rule to define persons with CA infection with *S. aureus*. This rule used the electronic records of all persons from whom MRSA or methicillin-susceptible *S. aureus* (MSSA) had been identified in cultures of soft tissue, pus, bone, or joints. Infections from patients who met the following electronic case definition were designated CA: 1) culture obtained as an outpatient or within the first 3 days of hospitalization, 2) no clinical culture with MRSA in the last 6 months, 3) no hospitalization or surgeries within 1 year, and 4) no hemodialysis. All other infections were defined as healthcare associated. Data for microbiology results, demographics, and recent surgery or hospitalization were linked by a unique patient identification number. Dialysis use was detected by the use of biochemical tests obtained around the time of dialysis or of hemodialyis-related ICD-9 procedure codes (39.27, 90945, 39.95, 90935, 54.98, 39.43, 39.42, or 38.95). Because the electronic data sources were complete for the period specified, absence of data for a patient was considered to be due to the absence of exposure, not missing data.

Using the electronic case definition and data repository, we randomly selected 100 patients with putative CA- and 100 with putative healthcare-associated *S. aureus* infections. The paper charts for these 200 patients were reviewed to validate the designations of CA- or healthcare-associated infection, by using the same criteria as for the electronic rule. To ensure blinding for manual chart reviews, all references to results of the electronic rule were removed from data collection instruments. Using information obtained from chart review as the standard, we determined sensitivity and specificity of the electronic rule and calculated agreement (κ statistic) between manual and electronic reviews. To ascertain data sources of most value in detecting healthcare exposures, we examined data tables required for each type of exposure and for coincident exposures to develop more parsimonious data requirements.

During the study period, 714 (386 MSSA and 328 MRSA) healthcare-associated and 1,222 (518 MRSA and 704 MSSA) CA infections occurred; all electronic data elements were available for all patient encounters that occurred within CCBHS. Sampling yielded 47 CA- and 52 healthcare-associated MRSA infections and 53 CA- and 48 healthcare-associated MSSA infections.

The electronic case definition performed well when compared with chart review. All 100 healthcare-associated infections identified electronically were confirmed by manual chart review as classified correctly. Among the 100 community-associated infections identified electronically, 3 (3%) were determined by chart review to have been misclassified: 2 patients had been hospitalized, and a third had surgery within the previous year, all outside CCBHS. The sensitivity of the electronic case definition for community association was 100%; specificity was 97%. The κ statistic was 0.97 (confidence interval [CI] 0.83–1.00), which indicated superior agreement between chart review and electronic rule. For misclassified cases, 1 infection was due to MRSA, and 2 were due to MSSA. The performance characteristics of the rule for CA-MRSA were sensitivity 100%, specificity 98.1%, and κ = 0.98 (CI 0.78–1.00).

The Table describes data elements required to detect healthcare exposures. The most data-intensive exposure to detect was hemodialysis, which required a search of laboratory and discharge diagnosis databases. Isolates of MRSA were designated healthcare-associated most commonly because of prior hospitalization (523 [73%] of 714) and date of culture (i.e., >3 days after hospital admission) (259 [36%] of 714). With the use of only admission/discharge and microbiology data, 28 patients (90%) who had undergone dialysis and 23 (85%) who had undergone surgery were identified. The use of only admission/discharge and microbiology data would have detected 707 patients, 99% of those who would have been detected by the full algorithm.

**Table:** Data sources for healthcare exposures

Healthcare exposure	Databases needed	Comments
Isolate obtained >3 d after admission	Microbiology laboratory	Microbiology table must contain patient registration date to compare with culture date
Hemodialysis	Discharge diagnoses, biochemistry laboratory	Biochemistry laboratory table must contain location of test to identify dialysis clinic
Prior hospitalization (within 1 y)	Admission, discharge, and transfer data	Query must be able to compare individual patients across admissions
Prior surgeries (within 1 y)	Operating room schedules	May not be readily available in many institutions
Prior isolation of MRSA* (within 6 mo)	Microbiology laboratory	Query must be able to compare individual patients across admissions

*MRSA, methicillin-resistant *Staphylococcus aureus*.

Our study had limitations. Chart review may have undercounted healthcare-associated factors and is dependent on clinician histories and documentation. However, retrospective review of paper charts is the principal method that infection control practitioners use to gather information. Also, this study was conducted at a single center that served a population that may have had difficulty seeking care elsewhere. For single hospitals or systems with a less captive population, electronic measures may not function as well until disparate systems can be integrated, i.e., at the level of health departments or through data sharing among regional health information organizations.

In conclusion, using easily accessible data from a computerized clinical data repository, we readily classified *S. aureus* and MRSA infections as CA or healthcare associated. Comparison of the electronic method with manual paper chart review demonstrated high agreement for MRSA (κ = 0.98). Additional review suggested that use of only 1 or 2 data sources efficiently detected prior healthcare exposures. A major dividend of increased use of information technology in healthcare is application of electronically stored data to improve public health surveillance.
